# Passive immunotherapy for Alzheimer’s disease: challenges & future directions

**DOI:** 10.1186/s12967-024-05248-x

**Published:** 2024-05-07

**Authors:** Ling Xiao Yi, Eng King Tan, Zhi Dong Zhou

**Affiliations:** 1 National Neuroscience Institute of Singapore, 11 Jalan Tan Tock Seng, Singapore, 30843 Singapore; 2 Department of Neurology, Singapore General Hospital, Outram Road, Singapore, 169608 Singapore; 3 Signature Research Program in Neuroscience and Behavioral Disorders, Duke-NUS Graduate Medical School Singapore, 8 College Road, Singapore, 169857 Singapore

## Abstract

Passive immunotherapy with specific antibodies targeting Amyloid β (Aβ) peptide or tubulin-associated unit (tau) protein has emerged as a promising therapeutic approach in Alzheimer’s disease (AD). However, in a recent phase III clinical study, Sperling et al. (N Engl J Med 10.1056/NEJMoa2305032, 2023) reported that solanezumab, a monoclonal antibody targeting Aβ peptide, failed to slow cognitive decline in AD patients. Previously, three other anti-Aβ antibodies, bapineuzumab, crenezumab, and gantenerumab, have also failed to show similar beneficial effects. In addition, three humanized antibodies targeting tau protein failed in their phase II trials. However, other anti-Aβ antibodies, such as lecanemab (a humanized mAb binds to soluble Aβ protofibrils), donanemab (a humanized mAb binds to insoluble, N-terminal truncated form of Aβ peptides) and aducanumab (a human mAb binds to the aggregated form of Aβ), have been shown to slow the decline of cognitive functions in early stage AD patients. The specific targets used in passive immunotherapy in these clinical trials may explain the divergent clinical outcomes. There are several challenges and limitations of passive immunotherapy using anti-Aβ antibodies and long term longitudinal studies are needed to assess their efficacy, side effects and cost effectiveness in a wider spectrum of subjects, from pre-dementia to more advanced dementia. A combination therapeutic approach using both anti-Aβ antibodies and other pharmaceutical agents should also be explored.

## Introduction

Alzheimer’s disease (AD) is an age-related neurodegenerative disorder characterized by progressive neurodegeneration with memory loss and cognitive impairment [2]. The pathological hallmarks of AD include formation of amyloid β (Aβ) plaques from aggregation of extracellular amyloid β (Aβ) peptides and deposition of intracellular neurofibrillary tangles (NFT) from accumulated tubulin associated unit (tau) protein [3]. According to the amyloid cascade hypothesis, the generation of Aβ peptides and formation of Aβ plaques is the pathogenic trigger for a pathological cascade, contributing to NFT formation and neurodegeneration in AD [4].

### Solanezumab trials in AD

Passive immunotherapy with specific anti-Aβ antibodies has emerged as a promising therapeutic approach. In a recent phase III clinical trial involving 1169 AD patients, Sperling et al. evaluated the therapeutic effects of solanezumab, a monoclonal antibody (mAb) targeting monomeric Aβ peptide, with negative outcomes. This is the third unsuccessful trial of solanezumab in AD, following two previously failures in 2014 and 2021 [5, 6]. Solanezumab is a mAb that recognizes and binds to the mid-domain of Aβ peptide to promote the clearance of soluble Aβ [7]. Previous in vivo studies on transgenic PDAPP mice AD model showed that administration of the m266 (the murine precursor of solanezumab) significantly elevated the concentrations of Aβ in plasma and inhibit the deposition of Aβ plaque in mice brains [8, 9]. The therapeutic effects of solanezumab were further supported by phase I and II trial investigations, as solanezumab treatment led to enhanced total (bound plus unbound) Aβ concentrations in cerebrospinal fluid (CSF) and plasma in a solanezumab dosage dependent manner in AD patients [10, 11]. In the current study, all participants were randomly divided into two groups to receive solanezumab or placebo (administered intravenously up to 1600 mg) every 4 weeks for 240 weeks [1]. However, the study showed that Solanezumab failed to alleviate the progressive cognitive decline in AD patients (based on Preclinical Alzheimer Cognitive Composite (PACC) score) and to down-regulate amyloid levels in AD patient brains (based on 18F-florbetapir positron-emission tomography (PET)), compared with participants who received a placebo [1]. Amyloid-related imaging abnormalities (ARIA) with microhemorrhage or hemosiderosis occurred in 29.2% of solanezumab group and 32.8% of placebo group respectively [1].

### Unsuccessful passive immunotherapy trials in AD

Two previous phase III clinical trials using solanezumab also showed negative results [1, 5, 6]. In the first phase III double-blinded trials (EXPEDITION 1 and EXPEDITION 2), mild-to-moderate AD patients received placebo or solanezumab (administered intravenously at 400 mg) every 4 weeks for 18 months. However, neither EXPEDITION 1 nor EXPEDITION 2 demonstrated significant improvements in primary outcomes, based on the assessment of cognitive subscale of the Alzheimer’s Disease Assessment Scale (ADAS-COG) and the Alzheimer’s Disease Cooperative Study–Activities of Daily Living scale (ADCS-ADL) [5]. Subsequently, Salloway et al. conducted a randomized, placebo-controlled, multi-arm trial to evaluate the beneficial effects of solanezumab in dominantly inherited AD in 2021. Similarly, solanezumab did not demonstrate any therapeutic effects to improve cognitive functions in AD patients, compared with the placebo group [6].

Besides solanezumab, other anti-Aβ antibodies also failed to show any beneficial effects to improve cognitive functions in AD in multiple phase III clinical trials. These unsuccessful anti-Aβ antibodies include bapineuzumab [12], a humanized mAb that targets the N-terminal of Aβ to clear away both fibrillar and soluble Aβ peptides; crenezumab [13], a humanized mAb that binds to monomeric and oligomeric Aβ peptide, as well as gantenerumab [14], a fully human mAb that targets aggregated Aβ. Moreover, the aggregation of p-tau protein and the formation of NFT is another pathological hallmark of AD. Similar to anti-Aβ interventions, passive immunotherapy with anti-tau antibodies targeting p-tau protein has been investigated. To date, three humanized mAbs targeting the N-terminal domain of tau protein, namely semorinemab, gosuranemab and tilavonemab, failed to slow the AD progression in phase II trial studies [15–17]. Furthermore, three anti-Aβ antibodies, namely, aducanumab [18] (a human mAb targets aggregated form of Aβ, approved by the US Food and Drug Administration (FDA) in 2021), lecanemab [19] (a humanized mAb binds to soluble Aβ protofibrils, approved by the US FDA in 2023) and donanemab [20] (a humanized mAb binds to insoluble, N-terminal truncated form of Aβ peptides), showed promising effects in trials to decelerate the progressive cognitive decline in early stage AD patients.

### Successful passive immunotherapy trials in AD

The therapeutic efficacy and safety of aducanumab were reported in 2022 with 3285 early tage AD patients separated into two random phase III trials (EMERGE with 1638 participants and ENGAGE with 1647 participants) [18]. All participants received aducanumab or placebo (administrated intravenously at 3, 6 and 10 mg per kilogram (kg) of body weight) every 4 weeks for 76 weeks. However, both EMERGE and ENGATE were terminated early due to the outcome of futility analysis, only 1812 (55.2%) participants in EMERGE and ENGAGE completed the study. A dose- and time-dependent reduction of amyloid level in AD patient brains and plasma hyperphosphorylated tau (p-tau) level (a downstream biomarkers specific to AD) were observed in both EMERGE and ENGAGE, compared with the placebo group. Treatment with low and medium dosages of aducanumab did not show any beneficial effects in AD patients, whereas high dosage (10 mg per kg of body weight) aducanumab administration alleviated the progressive cognitive decline in AD patients in EMERGE, but not in ENGAGE [18]. However, dose-dependent adverse effects were observed in both EMERGE and ENGAGE, including ARIA with edema, headache, brain microhemorrhages. nasopharyngitis, fall, localized superficial siderosis and dizziness [18].

In a multicenter, double-blind, phase III trial, 1795 early stage AD patients were grouped to receive lecanemab or placebo (administered intravenously at 10 mg per kg of body weight) randomly every 2 weeks for 18 months [19]. The study showed that lecanemab reduced the markers of amyloid in AD brains with slowed cognitive decline in early stage AD patients, compared with the placebo group [19]. However, lecanemab administration also led to unpleasant side effects, including infusion-related reactions, ARIA with cerebral microhemorrhages, cerebral macrohemorrhages, superficial siderosis and edema or effusions [19].

The therapeutic effects of donanemab were reported in a phase III trial in 2023, including 1736 early symptomatic AD patients with mild cognition impairment [20]. In the study AD patients received donanemab or placebo (administrated intravenously at 700 mg for the first three doses and 1400 mg thereafter) every 4 weeks for 72 weeks [20]. Donanemab treatment reduced the amyloid plaque level and slowed the cognitive decline in AD patients [20]. The donanemab treatment induced side effects included ARIA with edema or effusion in AD brains, infusion-related reactions and donanemab associated patient demise (3 deaths) [20]. Details of all reported anti-Aβ and anti-tau antibodies for passive immunotherapy trials in AD are summarized in Table 1.

**Table 1:** Summary of passive immunotherapy trials in AD

Antibody	Subclass	Origin	Epitope	Specificity	Patients	Duration	Dosage	Clinical outcomes		Side effects	References
Anti-Aβ mABs in phase III studies											
Aducanumab	IgG1	Fully human	AA3-6	Aggregated form of Aβ	EMERGE:1638 early AD patients. 543 participants received low dose aducanumab, 547 participants received high dose aducanumab and 548 participants received placebo ENGAGE:1647 early AD patients. 547 participants received low dose aducanumab, 555 participants received high dose aducanumab and 545 participants received placebo	Administered intravenously every 4 weeks over 76 weeks	Low dose: 3 and 6 mg/kg body weight High dose: 10 mg/kg body weight	Aducanumab reduced the levels of Aβ and p-tau in AD patients in both trials Only high dose of aducanumab slowed the cognitive decline in EMERGE study Both studies were terminated early due to the outcome of a futility analysis	In EMERGE: ARIA with edema Brain microhemorrhage Localized superficial siderosis Headache Nasopharyngitis Falls Dizziness In ENGAGE: ARIA with edema Brain microhemorrhage Localized superficial siderosis Headache Nasopharyngitis Falls Dizziness		[18]
Lecanemab	IgG1	Humanized	AA1-15	Soluble Aβ protofibrils	1795 early AD patients 898 participants received lecanemab and 897 participants received placebo	Administered intravenously every 2 weeks for 18 months	10 mg/kg;	Compared to the placebo group, Lecanemab slowed cognitive decline and reduced the Aβ plaque in early AD patients	Infusion-related reactions ARIA with edema or effusion ARIA with microhemorrhages and hemosiderin deposits Headache Fall Back pain Localized superficial siderosis		[19]
Donanemab	IgG1	Humanized	AA3-10	Insoluble, N-terminal region of Aβ peptides	1736 early symptomatic AD patients; 860 participants received donanemab and 876 participants received placebo	Administered intravenously every 4 weeks for 18 months	From 700 to 1400 mg;	Donanemab slowed cognitive decline and reduced Aβ plaque in early AD patients, compared to the placebo group	ARIA with edema or effusion ARIA with microhemorrhages and hemosiderin deposits Infusion-related reactions Death-related to treatment Headache Fall Localized superficial siderosis Dizziness		[20]
Bapineuzumab	IgG1	Humanized	AA1-5	N-terminal of Aβ	Trial 1: 1121 mild-to-moderate AD patients carry the ApoE4 allele. 673 participants received bapineuzumab and 448 participants received placebo Trial 2: 1331 mild-to-moderate AD patients without ApoE4 allele. 807 participants received bapineuzumab and 524 participants received placebo	Administered intravenously every 13 weeks for 78 weeks	Trial 1: 0.5 mg/kg body weight Every 13 weeks Trial 2: 0.5 and 1.0 mg/kg body weight	Bapineuzumab failed to improve cognitive function in AD patients	Trial 1: ARIA with edema or effusion Fall Headache Trail 2: ARIA with edema or effusion Fall Urinary tract infection Anxiety Headache Agitation		[12]
Crenezumab	IgG4	Humanized	AA13-24	Monomeric and oligomeric Aβ peptide	CREAD: 813 early AD patients. 404 participants receive crenezumab and 409 participants receive placebo CREAD2: 806 early AD patients. 407 participants receive crenezumab and 399 participants receive placebo	Administered intravenously every 4 weeks for 100 weeks	60 mg/kg body weight	Crenezumab failed to improve cognitive function in early AD patients	CREAD: Infusion-related reactions ARIA with edema ARIA with hemorrhage Fall Pneumonia Headache CREAD2: Infusion-related reactions ARIA with edema ARIA with hemorrhage Fall Pneumonia Headache		[13]
Gantenerumab	IgG1	Fully human	AA3-12, AA18-27	Aggregated Aβ	GRADUATE I: 985 early AD patients. 499 participants received gantenerumab, and 485 received placebo GRADUATE II: 980 early AD patients. 498 participants received gantenerumab, and 477 received placebo	Administered subcutaneously every 2 weeks for 116 weeks	Up to 1020 mg	Gantenerumab reduced Aβ plaque but failed to improve cognitive function in early AD patients	Across both trials: Injection-site reactions ARIA with edema Fall Headache Nasopharyngitis Dizziness Arthralgia		[14]
Solanezumab	IgG1	Humanized	AA16-26	Soluble monomeric Aβ peptide	EXPEDITION 1: 1012 mild-to-moderate AD patients. 506 participants received solanezumab and 506 participants received placebo EXPEDITION 2: 1040 mild-to-moderate AD patients. 521 participants received solanezumab and 519 participants received placebo	Administered intravenously every 4 weeks for 18 months	400 mg	Solanezumab failed to improve cognitive function and failed to reduce Aβ plaque in AD patients	Across both trials: ARIA with edema ARIA with hemorrhage Eye disorders Diarrhea Urinary tract infection Upper respiratory tract infection Nasopharyngitis Fall Headache Dizziness		[5]
Solanezumab	IgG1	Humanized	AA16-26	Soluble monomeric Aβ peptide	92 dominantly inherited AD patients. 52 participants received solanezumab and 40 participants received placebo	Administered intravenously every 4 weeks for 4 years	From 400 to 1600 mg	Solanezumab failed to improve cognitive function and failed to reduce Aβ plaque in AD patients	ARIA with edema ARIA with microhemorrhage Headache Nasopharyngitis Post-lumbar puncture syndrome Back pain Sinusitis		[6]
Solanezumab	IgG1	Humanized	AA16-26	Soluble monomeric Aβ peptide	1169 preclinical AD patients. 578 participants received solanezumab and 591 participants received placebo	Administered intravenously every 4 weeks for 240 weeks	From 400 to 1600 mg	Solanezumab failed to improve cognitive function and failed to reduce Aβ plaque in AD patients	ARIA with edema ARIA with microhemorrhage ARIA with superficial siderosis		[1]
Anti-tau mAbs in phase II studies											
Semorinemab	IgG4	Humanized	AA6-23	N-terminal tau	457 prodromal to mild AD, 94 participants received 1500 mg semorinemab, 136 participants received 4500 mg semorinema, 92 participants received 8100 mg semorinema and 135 participants received placebo	Administered intravenously every 2 weeks for the first three times and every 4 weeks thereafter for 73 weeks	1500, 4500 and 8100 mg	Semorinemab failed to slow the clinical progression in AD patients	Falls Nasopharyngitis Infusion-related reactions Arthralgia Hypertension Urinary tract infection Upper respiratory tract infection Anxiety Headache Dizziness		[15]
Gosuranemab	IgG4	Humanized	AA15-22	N-terminal tau	650 early AD patients, 118 participants received low-dose gosuranemab, 106 participants received intermediate-dose gosuranemab, 214 participants received high-dose gosuranemab and 214 particiapnts received placebo	Administered intravenously every 4 weeks or every 12 weeks for 78 weeks	Low dose 1: 125 mg every 4 weeks Low dose 2: 375 mg every 12 weeks Intermediate dose: 600 mg every 4 weeks High dose: 2000 mg every 4 weeks	Gosuranemab reduced the unbound N-terminal tau in cerebrospinal fluid but failed to slow the clinical progression in AD patients	Infusion-related reactions Falls Nasopharyngitis Arthralgia Headache, Diarrhea Constipation		[16]
Tilavonemab	IgG4	Humanized	AA25-30	Extracellular N-terminal tau	453 early AD patients, 108 participants received 300 mg tilavonemab, 116 participants received 600 mg tilavonemab, 113 participants received 2000 mg tilavonemab and 116 participants received placebo	Administered intravenously every 4 weeks for 96 weeks	300, 600 and 2000 mg	Tilavonemab failed to demonstrate therapeutic efficacy in AD patients	Microhaemorrhages Cerebral oedema Fall Headache Dizziness Upper respiratory tract infection Nasopharyngitis Urinary tract infection Diarrhea		[17]

### Limitations and future directions

The different and inconsistent outcomes with passive immunotherapy for AD suggest that the specific molecular targets and clinical trial methodology need to be reassessed. It is well known that the epitope and isotype of an antibody are crucial to their therapeutic efficacy. It has been proved that the isotype of anti-Aβ antibodies can influence Aβ plaque clearance and neuronal protections, whereas IgG2 antibodies against Aβ can be more effective in reducing neuropathology than IgG1 antibodies [21]. The isotype of most anti-Aβ antibodies is IgG1, which might be the underlying cause for the lower efficacy of some anti-Aβ antibodies in AD trials. Moreover, previous studies have suggested that anti-Aβ antibodies targeting the N-terminal region of Aβ peptides may invoke Aβ plaque clearance and neuronal protection [21]. The epitopes of three successful anti–Aβ antibodies (aducanumab, lecanemab and donanemab) all target the N-terminal region of Aβ peptides.

In addition, other factors, such as antibodies induced inflammatory responses and the penetration efficiency of antibodies through blood–brain barrier (BBB), also need to be addressed. The BBB prevents the penetration of most drugs, proteins and peptides from blood into brains, which is an existing challenge to be overcome [22]. While mAbs enter into patients via systemic administration (intravenous, intramuscular, or subcutaneous), it is unclear how much antibodies can pass through BBB to bind with Aβ peptides in the brain. Previous studies have shown that only approximately 0.1% of administrated mAbs can cross BBB, while the rest antibodies will be either metabolized in the liver or excreted via the kidney [23, 24]. The very limited BBB penetration efficiency of administrated antibodies can be a confounding variable in AD trials. Recent studies have suggested the alternative strategy to deliver drugs, proteins or peptides into brains via intranasal administration, which has advantages to systemic administrations [25]. Intranasal delivery of drugs can directly enter the brain, reducing drug exposures to peripheral organs and tissues, avoiding drug degradation in the circulation and enhancing the bioavailability of delivered drugs [26]. It was reported that daily intranasal administration of a novel PEI-conjugated R_8_-Aβ (25–35) peptide significantly reduced Aβ amyloid accumulation and ameliorated the memory deficits in PS-1/APP mice AD model [27]. The intranasal delivery of full-length anti-Nogo-A antibody could promote growth and compensatory sprouting of corticofugal projections and enhance functional recovery in a rat stroke model [28]. Moreover, intranasal delivery of insulin improved cognitive functions in AD patients and human subjects with amnestic mild cognitive impairment [29]. These findings support the feasibility of passive immunotherapy in AD via intranasal administration [28, 29]. Nevertheless, there are still several disadvantages of intranasal administration. AD is a chronic disorder, repeated intranasal administration may cause irreversible damage to the nasal epithelium, nasal mucosa and nerves in the cavity since their surface area is limited [30]. Future investigations are warranted to evaluate the safety, efficacy and therapeutic effects of intranasal administration in passive immunotherapy in AD and other human neurodegenerative diseases.

The repeated injection of antibodies as exogenous proteins may induce immune response and generate anti-drug antibodies (ADA) against administrated antibodies, which can interact, neutralize and down-regulate the levels of administrated antibodies for passive immunotherapy [31, 32]. Adalimumab is an antibody targeting tumor necrosis factor-α (TNF-α) which has been used effectively in passive immunotherapy for rheumatoid arthritis. Previous studies have showed higher serum levels of ADA in patients after adalimumab treatment, which is linked to impaired therapeutic effects of adalimumab treatment [33]. The formation of ADA and impact on therapeutic efficacy have also been reported in patients with tumors and inflammatory disorders [34]. The formation of ADA has also been identified in AD patient serum in passive immunotherapy trials with crenezumab (an anti-Aβ antibody), tilavonemab (an anti-tau antibody) and solanezumab [13, 17, 35]. The potential adverse effects of ADA in passive immunotherapy in AD should be further investigated in future studies.

Besides ADA, other factors may be involved in influencing the outcomes of passive immunotherapy trials. The formation and deposition of antigen–antibody complexes in multiple organs and tissues can trigger pathological inflammatory response via stimulating complement cascade and Fc receptors in immune cells [36]. Given that the therapeutic anti-Aβ and anti-tau antibodies can pass through BBB in passive immunotherapy and penetrate into disease plaques to bind with Aβ peptides, the Aβ peptides-antibody complex needs to be cleared from patient brains in time. Otherwise, antigen–antibody complex deposition may trigger inflammatory response to aggravate neurodegeneration in affected areas. The presence of anti-Aβ autoantibodies in CSF have been reported to induce amyloid angiopathy-related inflammation in patients with focal neurological symptoms and cognitive impairment [37]. Furthermore, numerous studies suggest that autoantibodies target cell surface, intracellular and extracellular proteins. These can trigger auto-immune response, resulting in neuronal injury and neurodegeneration [38]. The potential adverse effects induced by antigen–antibody complex formation and deposition during passive immunotherapy should be taken into consideration in future studies.

It is known that neuronal debris and toxic proteins need timely clearance through phagocytosis of microglia and astrocytes to maintain brain homeostasis [39]. In passive immunotherapy in AD, the formation of antigen–antibody complexes will need phagocytotic clearance by microglia and astrocytes. However, recent observations showed that the phagocytosis capacities of microglia and astrocytes are significantly impaired in AD. In PS1-APP mice the phagocytosis capacity of microglia was significantly reduced with down-regulated expression of scavenger receptors and pathogenic protein degrading enzymes [40]. The astrocytes from AD mice have a lower capacity to scavenge the extracellular Aβ, as Aβ peptides directly suppress the phagocytosis capacity of astrocytes [41]. The impaired phagocytosis of microglia and astrocytes in AD will disturb the clearance of antigen–antibody complexes in passive immunotherapy, leading to potential accumulation and deposition of antigen–antibody complex, and subsequent inflammatory response and neuron injury. So far three anti-Aβ antibodies, aducanumab, lecanemab and donanemab, have showed some beneficial effects only in early stage AD patients. The phagocytosis capacity of microglia and astrocytes may be higher in early stage AD patient brains, which may be a favorable factor for the aducanumab, lecanemab and donanemab trials as they target the early stage AD patients.

Studies suggest that the phagocytosis capacities of microglia and astrocytes can be enhanced by modulation of peroxisome proliferator-activated receptor γ (PPARγ) and AXL receptor tyrosine kinase pathways. It is reported that in PS-1/APP AD mice model the phagocytosis capacity of microglia can be enhanced by small molecular PPARγ modulator, DSP-8658, with up-regulated expression of scavenger receptor in microglia cells [42]. Genistein can also activate PPARγ signaling pathway to promote Aβ clearance, reduce Aβ plaques and improve cognitive function in AD mice model [43]. The intranasal administration of recombinant mouse growth arrest-specific 6 (rmGas6) protein, a specific ligand of AXL receptor, activates AXL receptor tyrosine kinase and promotes the conversion of astrocytes into phagocytic phenotype and enhances phagocytic capacity of astrocytes in traumatic brain injury mice model [44]. In addition, administration of jujuboside A or ganoderic Acid A can activate AXL signaling pathway to promote Aβ clearance and ameliorate cognitive deficiency in AD mice model [45, 46]. Therefore combination therapy including passive immunotherapy antibodies and modulators of PPARγ and /or AXL pathways may achieve better therapeutic effects in AD. However, all PPARγ and AXL pathways modulators have not been validated in human. Furthermore, the AXL receptor tyrosine kinase is a biomarker and therapeutic target associated with tumor growth and poor prognosis in cancer [47]. Further studies on regulations of phagocytic capacities of human microglia and astrocytes by PPARγ and AXL signaling pathways modulators to enhance therapeutic effects of AD passive immunotherapy are needed.

## Conclusions

Passive immunotherapy trials in AD have not produced consistent results, with disappointing results from the studies using solanezumab, bapineuzumab, crenezumab, and gantenerumab. Though aducanumab, lecanemab and donanemab show some promising results in early stage AD patients [15–17], long term follow-up data and studies in middle and late stage AD patients will be needed. To date, only lecanemab and aducanumab have received FDA approval. The prevalence and severity of side effects (such as infusion-related reactions, ARIA with cerebral microhemorrhages, cerebral macrohemorrhages, superficial siderosis, etc.) will be clearer with more widespread clinical use. The poor antibody penetration across BBB, ADA neutralization of administrated antibodies, brain inflammation triggered by antigen–antibody complex deposition as well as adverse effects induced by impairment of phagocytosis capacities of microglia and astrocytes in AD brains can be challenges for AD passive immunotherapy studies. The intranasal administration of antibodies to avoid BBB obstacle can be an alternative delivery strategy to increase their concentrations in the brain. Combination strategies such as passive immunotherapy antibodies with pharmaceutical agents that can promote microglia and astrocytes phagocytosis activities may be potentially more effective and should be further explored.

## Data Availability

Not applicable.
